# High Temperature Oxidation Behavior of Additive Manufactured Ti6Al4V Alloy with the Addition of Yttrium Oxide Nanoparticles

**DOI:** 10.3390/ma17112544

**Published:** 2024-05-24

**Authors:** Qiang Wang, Pu Song, Wenjuan Niu, Nan Li, Ning Hu

**Affiliations:** 1 School of Metallurgical Engineering, Xi’an University of Architecture and Technology, Xi’an 710055, China; pusong0522@163.com (P.S.); nanlin@xauat.edu.cn (N.L.); huning@xauat.edu.cn (N.H.); 2 Shaanxi Province Metallurgical Engineering Technology Research Center, Xi’an 710055, China

**Keywords:** additive manufacturing, Ti6Al4V, oxidation behavior, surface, microhardness

## Abstract

Titanium alloys face challenges of high temperature oxidation during the service period when used as aircraft engine components. In this paper, the effect of Y_2_O_3_ addition on the oxidation behavior and the microstructural change of the Ti6Al4V alloy fabricated by selective laser melting (SLM) was comprehensively studied. The results show that the surface of the Ti6Al4V alloy is a dense oxide layer composed of TiO_2_ and Al_2_O_3_ compounds. The thickness of the oxide layer of the Ti6Al4V increased from 59.55 μm to 139.15 μm. In contrast, with the addition of Y_2_O_3_, the thickness of the oxide layer increased from 35.73 μm to 80.34 μm. This indicates that the thickness of the oxide layer formation was a diffusion-controlled process and, therefore, the thickness of the oxide layer increased with an increase in temperature. The Ti6Al4V-1.0 wt.% Y_2_O_3_ alloy exhibits excellent oxidation resistance, and the thickness is significantly lower than that of the Ti6Al4V alloy. The oxidation kinetics of the Ti6Al4V and Ti6Al4V-1.0 wt.% Y_2_O_3_ alloys at 600 °C and 800 °C follows a parabolic rule, whereas the oxidation of the Ti6Al4V and Ti6Al4V-1.0 wt.% Y_2_O_3_ alloys at 1000 °C follows the linear law. The average microhardness values of Ti6Al4V samples after oxidation increased to 818.9 ± 20 HV_0.5_ with increasing temperature, and the average microhardness values of the Ti6Al4V-1.0 wt.% Y_2_O_3_ alloy increases until 800 °C and then decreases at 1000 °C. The addition of Y_2_O_3_ shows a significant improvement in the microhardness during the different temperatures after oxidation.

## 1. Introduction

Selective Laser Melting (SLM) is one of the most developed additive manufacturing (AM) methods for metallic materials. Compared with the traditional manufacturing method, the unique layer-wise process has a high freedom of customization in fabricating near net-shaped components with complex geometries [1,2]. Due to the advantages of low density, high specific strength, and excellent corrosion resistance, titanium and its alloys have been widely used in the fields of aerospace, aviation, and biomedical industries [3,4,5]. In particular, as a type of dual phase alloy, the Ti6Al4V has good mechanical properties. However, the inadequate oxidation resistance at elevated temperatures limits the use of Ti6Al4V alloys as vital strength members in advanced heat engines [6]. The utilization of titanium alloys is typically restricted to temperatures under 600 °C because of the oxidation and the diffusion of oxygen [7]. The method to improve efficiency is to increase the pressure in the engines, and the temperature in the engines also increases. Usually, jet engines have a relatively short period of time at the maximum during a normal flight. Considering that jet engines operate for thousands of flight hours during their total life, if each such short period of time is increased over a period of time, the time at the maximum temperature becomes significant [8]. The performance of titanium alloys deteriorates significantly with increasing temperature, resulting in reduced resistance to hot air. This is a common phenomenon for metals oxidized at high temperatures, which greatly influences their properties, surface finish, and service life [9]. Moreover, α-Ti has a very high affinity for oxygen at elevated temperatures, leading to the formation of an oxygen-rich and brittle layer, which has an adverse effect on mechanical properties and increases the sensitivity of component cracking; the performance of the titanium alloy will be significantly degraded, and the resistance will be limited [8]. In addition, the oxidation of titanium alloys at high temperatures in air not only produces oxide scales, but it also leads to solid solution hardening of the surface, which results in the formation of hardened zones [10].

The common method used to improve the oxidation resistance of titanium alloys mainly includes surface treatment and alloying by the addition of elements to improve the properties of the alloy, such as Mn, Nb, B, and Y [11,12]. Some common surface treatment methods mainly include coatings and laser shock processing (LSP) [13]. Compared with surface treatments, alloying is more suitable for an AM process. Studies have shown that the addition of rare earth elements is beneficial for improving oxidation-resistance, in particular of yttrium (Y) and its oxide. For example, Luan et al. [14] added a small amount of B and Y to the as-cast Ti6Al4V alloy. The results showed that B is beneficial not only to grain refinement but also to grain boundary enhancement, while Y can effectively improve the oxidation resistance by slowing down oxidation kinetics. Zhao et al. [15] found that the addition of Y can improve the isothermal and cyclic oxidation resistance of the Ti-45Al-8Nb alloy. However, due to the increase of lattice defects, excessive Y addition is not conducive to oxidation resistance. Kenel et al. [16] reported that the addition of Y_2_O_3_ can increase the oxidation resistance of a spark plasma sintered (SPS) and direct metal deposited (DMD) Ti-45Al-3Nb alloy up to 800 °C, and compared with that of original alloys, the parabolic growth constants for oxide are decreased by 49% and 75% for SPS and DMD alloys, respectively. Moreover, nanoparticles have the characteristics of high specific surface area and small grain size, and the increase of interfacial energy can contribute to improve the stability and oxidation resistance [17,18].

The present study aims to investigate the effect of adding Y_2_O_3_ nanoparticles on the oxidation behavior of SLM Ti6Al4V alloys under different oxidation temperatures. The change of oxide morphology, the formation mechanism of oxide, and oxidation kinetics will be comprehensively discussed.

## 2. Materials and Experiments

### 2.1. Powder Materials

**Figure 1:**
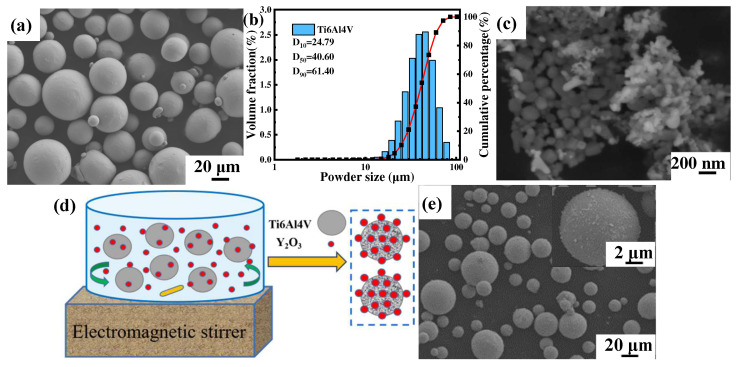
SEM images of powder morphology (**a**) Ti6Al4V powder, (**b**) Ti6Al4V particle size distribution, (**c**) Y_2_O_3_ powder, (**d**) Schematic diagram of electromagnetic stirring of composite powders, (**e**) Ti6Al4V-1.0 wt.% Y_2_O_3_ composite powder and the enlarged surface (inset).

The gas atomized near-spherical commercial Ti6Al4V powder (Avimetal AM Tech, Beijing, China) with a particle size of 15–53 μm and the irregularly shaped Y_2_O_3_ (Jingrui New Material, Xuancheng, China) with an average particle size of 30 nm were used as raw materials, and the morphology is shown in Figure 1a,c, respectively. The particle size distribution of Ti6Al4V powder measured by a laser scattering particle size distribution analyzer (Mastersizer 2000, Malvern Instruments, Malvern, UK) showed a normal distribution between 24.97 μm (D10) and 61.40 μm (D90), and the average volume diameter was 40.60 μm (D50), as shown in Figure 1b. The content of the added nanoparticles was set to 1.0 wt.%, and the composite powders were prepared by electromagnetic stirring and dispersion treatment, followed by drying at 70 °C for 10 h in a vacuum drying oven, as schematically shown in Figure 1d. The morphology of composite powders is shown in Figure 1e, and it is noticed that the irregularly shaped Y_2_O_3_ nanoparticles were uniformly distributed on the surface of spherical Ti6Al4V powders.

### 2.2. SLM Process

**Figure 2:**
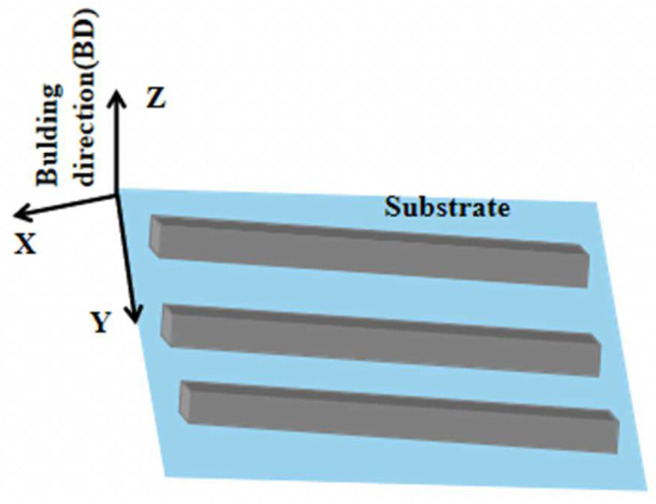
Schematic illustration of the SLM-fabricated samples.

The SLM process was conducted in a Concept Laser M2 machine equipped with a 100 W fiber laser. The process parameters were set as laser power 100 W, focusing spot diameter 50 μm, scanning speed 800 mm s^−1^, scanning line spacing 80 μm, each powder layer thickness 25 μm. The laser scanning method was a random island scanning strategy to reduce the accumulation of heat and residual stress in the part of the component, and high purity argon was used as the protective gas. The rectangular samples of 46 × 10 × 20 mm^3^ (X × Y × Z) were prepared without preheating the substrate, and the schematic illustration of the SLM-fabricated samples is shown in Figure 2.

### 2.3. Oxidation Performance Test

The oxidation test was carried out in an SSX2-8-16 C box-type resistance furnace in static laboratory air, and the temperature fluctuation range was 5 °C. The samples were heated to 600 °C, 800 °C, and 1000 °C, holding 24 h. The samples were cut into a size of 6 × 6 × 3 mm^3^ blocks, and the surface, cross-sectional morphology, and chemical composition of the oxide film after oxidation were analyzed. Two parallel specimens for each composition were prepared to show the reproducibility of the tests. The samples were weighed at intervals of 4 h. The mass of each sample before and after oxidation at different time periods was determined by an electronic analytical balance with an accuracy of 0.1 mg.

### 2.4. Characterization Methods

The samples before and after oxidation were examined, and phase identification was conducted by using an X-ray diffractometer (XRD, BrukerD8, Karlsruhe, Germany) with a Cu-Kα radiation source, 40 KV and 40 mA, a step size of 0.02°, and a scanning speed of 5°/min. The microstructure was characterized through a scanning electron microscope (SEM, Gemini FE-SEM 300, ZEISS, Jena, Germany) equipped with an energy dispersive spectrometer (EDS).

According to ASTM E384 standard [19], the microhardness of the sample was measured by a Vickers hardness tester (HXD-2000TMC/LCD, Taiming, Shanghai, China) with a load of 500 gf and a residence time of 15 s.

## 3. Results and Discussion

### 3.1. Oxide Formation at Different Temperatures

**Figure 3:**
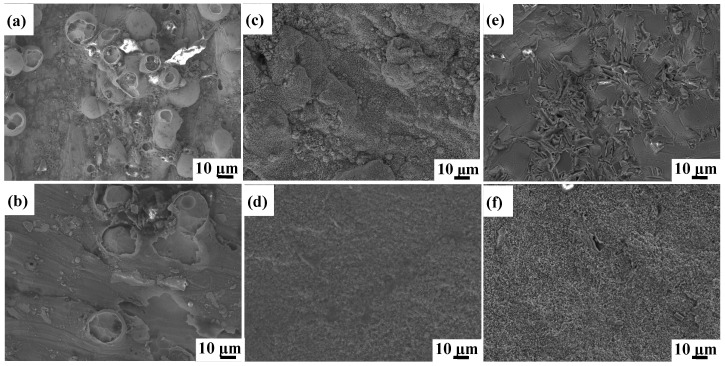
SEM of surface morphologies of Ti6Al4V and Ti6Al4V-1.0 wt.% Y_2_O_3_ samples after oxidation for 24 h (**a**,**b**) 600 °C, (**c**,**d**) 800 °C, (**e**,**f**) 1000 °C.

The oxidized surface morphologies of as-printed Ti6Al4V and Ti6Al4V-1.0 wt.% Y_2_O_3_ samples at 600 °C, 800 °C, and 1000 °C for 24 h are shown in Figure 3. The morphologies of Ti6Al4V and Ti6Al4V-1.0 wt.% Y_2_O_3_ samples heated at 600 °C are shown in Figure 3a and Figure 3b, respectively. The oxidized surface of the Ti6Al4V exhibits a large number of spherical oxide particles with random distributions, while the Ti6Al4V-1.0 wt.% Y_2_O_3_ sample has few numbers of oxide particles. When samples were heated at 800 °C, the clusters of aggregated small particles are observed on the surface of the Ti6Al4V (Figure 3c), which creates increased surface roughness. In contrast, the Ti6Al4V-1.0 wt.% Y_2_O_3_ alloy exhibits a finely grained and dense oxide layer with a smooth and flat surface (Figure 3d). With a further increase of the oxidation temperature to 1000 °C, there are large grained oxide clusters on the sample surface of the Ti6Al4V (Figure 3e). For the Ti6Al4V-1.0 wt.% Y_2_O_3_ sample (Figure 3f), the surface oxide still remains in a finer state but shows a relatively rough morphology with a few random large oxide crystals, compared with the sample at 800 °C.

It can be seen that as the temperature increases from 600 °C to 1000 °C, the oxide on the surface of the Ti6Al4V sample continues to grow, showing a water ripple pattern at 1000 °C, which make the oxides on the surface of the Ti6Al4V-1.0 wt.% Y_2_O_3_ sample become denser. It is evident that the surface oxide layer on the Ti6Al4V sample continues to thicken. At the early stage of oxidation, the growth rate of the oxide film on the alloy surface was fast, while with the extension of time, the oxide film was gradually thickened and the oxidation rate began to decrease. After the addition of nanoparticles, the smaller size of the nanoparticles was filled in between the gaps of the Ti6Al4V, resulting in a tighter arrangement between the atoms. With the temperature increase from 600 °C to 1000 °C, the oxide film changes have few numbers of dispersion particles to densification. The dense oxide surface is observed at 800 °C, and the oxide surface begins to become rough at 1000 °C. The oxide surface is mainly composed of Al_2_O_3_ compounds as revealed by XRD analysis results in Section 3.2. Therefore, the addition of Y_2_O_3_ will form a denser oxide film to protect the matrix, so that most of the unfavorable conditions are prevented outside the oxygen diffusion layer, which leads to the improvement of oxidation resistance.

**Figure 4:**
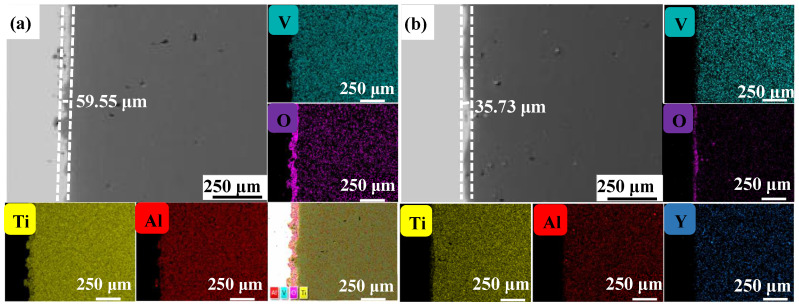
The cross-sections of as-printed samples’ oxidation at 600 °C for 24 h and their EDS mappings (**a**) Ti6Al4V and (**b**) Ti6Al4V-1.0 wt.% Y_2_O_3_ (with relative element distributions of Ti, Al, V, O, and Y).

**Figure 5:**
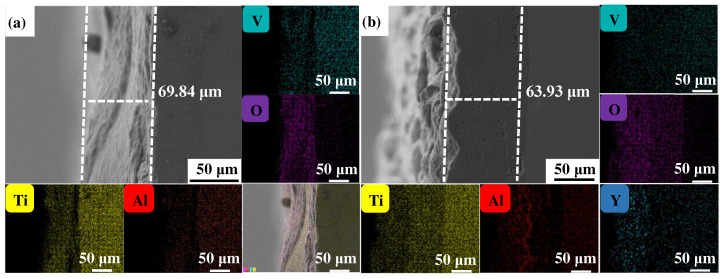
The cross-sections of as-printed samples’ oxidation at 800 °C for 24 h and their EDS mappings (**a**) Ti6Al4V and (**b**) Ti6Al4V-1.0 wt.% Y_2_O_3_ (with relative element distributions of Ti, Al, V, O, and Y).

**Figure 6:**
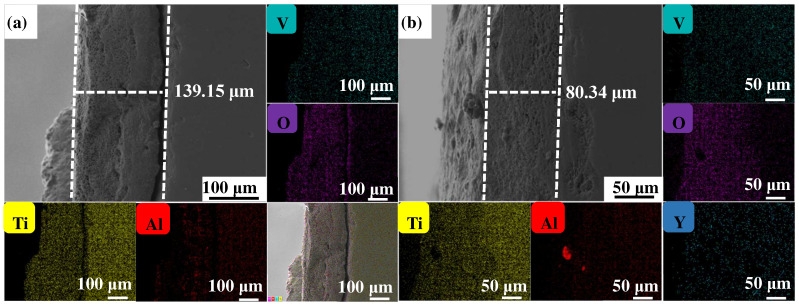
The cross-sections of as-printed samples’ oxidation at 1000 °C for 24 h and their EDS mappings (**a**) Ti6Al4V and (**b**) Ti6Al4V-1.0 wt.% Y_2_O_3_ (with relative element distributions of Ti, Al, V, O, and Y).

Figure 4, Figure 5 and Figure 6 show the cross-sectional microstructure and relevant element distributions of the Ti6Al4V and Ti6Al4V-1.0 wt.% Y_2_O_3_ samples at 600 °C, 800 °C, and 1000 °C, respectively. A narrow oxygen-rich region on the surface, indicated by the energy spectrum analysis, reveals the distribution of oxide layers, and the thickness is summarized in Table 1. It is noticed that the thickness of the oxide layer of the original Ti6Al4V increased from 59.55 μm to 139.15 μm. In contrast, with the addition of Y_2_O_3_, the thickness of the oxide layer increased from 35.73 μm to 80.34 μm. The thickness of the oxide layer of the Ti6Al4V-1.0 wt.% Y_2_O_3_ sample is consistently smaller than that of the Ti6Al4V, which is attributed to the addition of Y_2_O_3_ that suppressed the oxidation process. The temperature increase from 600 °C to 1000 °C and the thickness of the oxide layer significantly affect the growth on the Ti6Al4V and Ti6Al4V-1.0 wt.% Y_2_O_3_ samples. This phenomenon is attributed to the enhanced kinetics of the oxidation reaction at higher temperatures. In addition, the diffusion rate of oxygen atoms and titanium ions increases at high temperatures, leading to a faster rate of oxide formation. Consequently, the oxide layer thickens over time as the sample is exposed to higher temperatures. The results in Figure 4, Figure 5 and Figure 6 and Table 1 demonstrate the dimensions of the formed layer, which show an increasing trend due to the accelerated kinetics of the oxidation reaction as the temperature increases. This is due to the addition of Y_2_O_3_, which suppressed the oxidation process. In the case of the original Ti6Al4V alloy sample, at 1000 °C there is obvious delamination between the oxide layer and the matrix (Figure 6a), while intimate bonding can be seen in the Ti6Al4V-1.0 wt.% Y_2_O_3_ sample (Figure 6b). This is because the thermal expansion coefficients between the matrix and the oxide film do not match, thus leading to the generation of internal stresses [20,21]. The coefficients of the thermal expansion of TiO_2_ are 8.0 × 10^−6^ K^−1^ and those of Al_2_O_3_ are 6.5 × 10^−6^ K^−1^ [22]. The EDS mapping shows that there is a narrow oxygen-enriched zone in the oxide layer on the surface of the Ti6Al4V and Ti6Al4V-1.0 wt.% Y_2_O_3_ alloys.

### 3.2. XRD Phase Analysis

**Figure 7:**
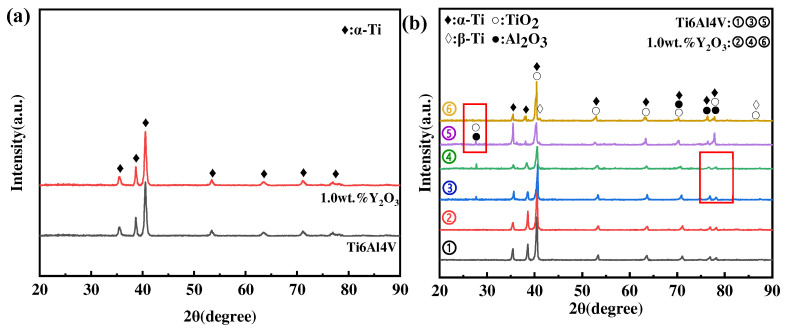
XRD of as-printed Ti6Al4V and Ti6Al4V-1.0 wt.% Y_2_O_3_ before and after oxidation at different temperatures for 24 h (**a**) as-printed samples, (**b**) after oxidation samples (➀, ➁ is 600 °C, ➂, ➃ is 800 °C, ➄, ➅ is 1000 °C).

Figure 7a shows the XRD results of phases in as-printed Ti6Al4V and Ti6Al4V-1.0 wt.% Y_2_O_3_ specimens before the oxidation experiment, and most of the peaks mainly belong to the α-Ti phase, while no obvious peaks of the β-Ti phase appear, due to a low volume content of it. The XRD results of the surface phases after oxidation at different temperatures are shown in Figure 7b. It shows that the oxide film on the surface of Ti6Al4V and Ti6Al4V-1.0 wt.% Y_2_O_3_ alloys oxidized at 600 °C is mainly composed of α-Ti and TiO_2_ (rutile-type), and this corresponds well with the phenomenon in Figure 7a,b, where discontinuous oxide films were exposed on the surface. As the temperature increased to 800 °C, the diffraction peaks’ intensity of TiO_2_ increased, and, meanwhile, a small amount of Al_2_O_3_ phase was identified, which can be explained thermodynamically by the fact that α-Al_2_O_3_ is more favorable to be produced at temperatures higher than 700 °C. However, the α-Al_2_O_3_ has a hexagonal structure with lattice constants of a = 4.75 Å, c = 12.97 Å, which differs from the lattice constant rutile [7]. The previous study also reported that Al_2_O_3_ can only nucleate and grow independently through the diffusion of Al atoms from the substrate to the scale surface via the TiO_2_ film [23,24]. Importantly, by comparing the XRD spectrum of lines ➂ and ➃ (the marked region), it is noted that the diffraction peak of Al_2_O_3_ is weaker for the Ti6Al4V-1.0 wt.% Y_2_O_3_ alloy, which indicates that the addition of Y_2_O_3_ suppressed the diffusion of Al atoms and improved the oxidation resistance. When the temperature went up to 1000 °C, the oxidized surface consists mainly of TiO_2_ and Al_2_O_3_. It is also noted that the oxide phase was dominated by TiO_2_ in the sample of the Ti6Al4V-1.0 wt.% Y_2_O_3_ alloy (line ➅) with a reduced amount of Al_2_O_3_, evidenced by the reduced intensity (the marked region between lines ➄ and ➅). In addition, the β-Ti phase is identified at 1000 °C, which is higher than the transition temperature of about 980 °C [25]. In addition, only minor differences in the diffraction peaks’ intensity were observed after oxidation at the same temperature.

### 3.3. Oxidation Kinetics of Alloy

**Figure 8:**
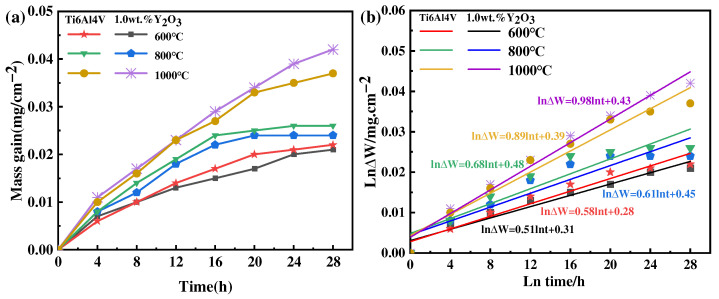
Thermogravimetric results of the Ti6Al4V-1.0wt.% Y_2_O_3_ alloy at 600 °C, 800 °C, and 1000 °C (**a**) weight gain curves and (**b**) lnΔW-lnt plots.

The results of the oxidation kinetics of as-printed Ti6Al4V and Ti6Al4V-1.0 wt.% Y_2_O_3_ samples at 600 °C, 800 °C, and 1000 °C are shown in Figure 8a. It can be seen that the oxidation weight gain rate of the Ti6Al4V-1.0 wt.% Y_2_O_3_ sample is slightly lower than the Ti6Al4V sample. At 600 °C and 800 °C, the oxidation kinetics curves are characterized by fast and then slow. When the temperature increases to 800 °C, as identified by the XRD analysis, the generation of the Al_2_O_3_ phase plays a protective role, and its kinetic curve shows more gentle characteristics. When the temperature reaches 1000 °C, its kinetic curve shows steeper characteristics. With the temperature increased, the oxidation rate of the Ti6Al4V alloy increases, and the thickness of the oxide film also increases with the extension of time, as revealed in Figure 4, Figure 5 and Figure 6. The slope of the oxidation kinetic curve gradually increases with the increase of temperature. At the initial oxidation stage at 600 °C, the weight gain rapidly increases around 8 h, and then the weight change of the sample becomes relatively stable. When the temperature is 800 °C, the weight gain rapidly increases within the first 12 h. When the temperature is 1000 °C, the weight gain rapidly increases throughout the whole process, which is attributed to the high temperature accelerating the movement of atoms and the rate of chemical reactions. This kind of phenomenon is consistent with the previous study on the oxidation behavior of the Ti6Al4V alloy in a temperature range of 650 °C to 850 °C, by Du et al. [26].

In general, the oxidation kinetics equation can be used to represent the oxidation behavior of metals at elevated temperatures. The oxidation kinetics results of the Ti6Al4V and Ti6Al4V-1.0 wt.% Y_2_O_3_ samples at 600 °C, 800 °C, and 1000 °C can be expressed. The curve of the weight gain versus the square root of time can be fitted with a straight line: see Equation (1) [27]:

(1) $$(∆W)=Kt^{n}$$

where K is the rate constant, ∆W represents the weight gain increase, and t is the oxidation time. Based on the data in Figure 8a, the fitted curve of ln∆W versus lnt is shown in Figure 8b, and the fitting linear slope of the lnΔW − lnt diagram is the value of exponent n. The predicted values are always slightly higher than the measured values.

The results show that the values of n of the Ti6Al4V and Ti6Al4V-1.0 wt.% Y_2_O_3_ alloys oxidation at 600 °C are 0.58 and 0.51, respectively, which are close to the value of n, the parabolic rate constant (0.5). With the temperature increased to 800 °C, the values of the Ti6Al4V and Ti6Al4V-1.0 wt.% Y_2_O_3_ alloys are 0.68 and 0.61, respectively, which are close to the value of the parabolic rate constant (0.5). It can be seen that the oxidation process at 600 °C and 800 °C follows the parabolic rule. The oxidation tests conducted in flowing synthetic air at 600 °C and 800 °C demonstrate that Y_2_O_3_ addition improves the oxidation resistance of Ti6Al4V alloys. Therefore, the process is controlled by oxygen diffusion [28]. This is due to the oxygen diffusion by the Ti6Al4V alloy during oxidation, which is used for the formation of oxide film. The oxygen diffusion distance increases with the increase of oxide film thickness. In other words, oxygen passing through the oxide layer is the control step of the oxidation rate of the material [29]. When the temperature increases to 1000 °C and the value of n is 0.89 for Ti6Al4V and 0.98 for Ti6Al4V-1.0 wt.% Y_2_O_3_, which are close to 1, the oxidation process conforms to the linear law. The interfacial reaction between oxygen and the oxide film/substrate is the control step of material oxidation behavior, which is related to the generation of cracks for Ti6Al4V [27]. In addition, the small cracks in the oxide form a connection with their increase, thus creating a larger path for the diffusion of oxygen into the matrix. In this case, the oxide film is considered to lose its protective effect on the substrate [30,31].

### 3.4. Vickers Microhardness Measurements

**Figure 9:**
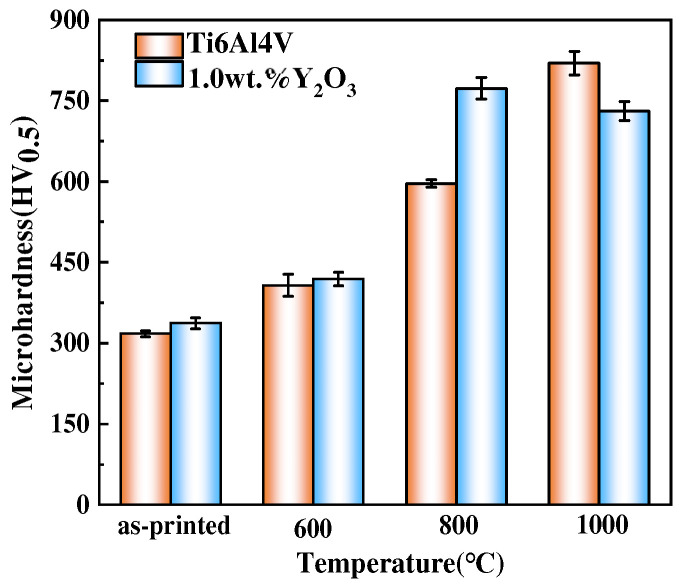
Microhardness test results of the Ti6Al4V and Ti6Al4V-1.0 wt.% Y_2_O_3_ alloy after oxidation.

The average microhardness results of the Ti6Al4V and Ti6Al4V-1.0 wt.% Y_2_O_3_ samples as-printed condition and after oxidation at different temperatures is shown in Figure 9. The average microhardness values of as-printed Ti6Al4V and Ti6Al4V-1.0 wt.% Y_2_O_3_ alloys are 317 ± 6 HV_0.5_ and 337 ± 10 HV_0.5_, respectively. It can be seen that the average microhardness value increases slightly after adding Y_2_O_3_ nanoparticles. After oxidation at 600 °C, the average microhardness value of the Ti6Al4V alloy is 403.2 ± 15 HV_0.5_ and the Ti6Al4V-1.0 wt.% Y_2_O_3_ alloy is 420 ± 12 HV_0.5_. Through oxidation at 800 °C, the average microhardness value of both the Ti6Al4V and Ti6Al4V-1.0 wt.% Y_2_O_3_ alloys increased significantly, to 588.3 ± 13 HV_0.5_ and 763 ± 20 HV_0.5_, respectively. After oxidation at 1000 °C, the average microhardness value of the Ti6Al4V alloy continued to increase, and the value is 818.9 ± 20 HV_0.5_. However, the average microhardness value of the Ti6Al4V-1.0 wt.% Y_2_O_3_ alloy decreased to 731.3 ± 15 HV_0.5_. The addition of Y_2_O_3_ slightly improved the microhardness value of the as-printed sample. With the temperature increased, in comparison to the as-printed samples, the surface microhardness value of Ti6Al4V shows an increasing trend. However, the surface microhardness value of the Ti6Al4V-1.0 wt.% Y_2_O_3_ alloy increases until 800 °C and then decreases at 1000 °C, which can be explained by the competition mechanism. The change in microhardness is mainly attributed to the heterogeneity quality and the oxide surface layer hardening occurring [7], which is also affected by the increase in the oxidation temperature and the diffusion of oxygen from the outside of the oxide layer [32].

## 4. Conclusions

(1) The surface of the Ti6Al4V oxidized at 600 °C, which showed a large number of spherical particles in a randomly dispersed arrangement, and few numbers of particles appeared on the surface of the Ti6Al4V-1.0 wt.% Y_2_O_3_ sample. With the temperature increase from 800 °C to 1000 °C, there were a few particle aggregates. In contrast, the Ti6Al4V-1.0 wt.% Y_2_O_3_ sample had finer particles in the oxide layer, with a smoother and flatter surface arrangement.
(2) With the temperature increase from 600 °C to 1000 °C, the thickness of the oxide layer of the Ti6Al4V and Ti6Al4V-1.0 wt.% Y_2_O_3_ increased. The thickness of the oxide layer of the Ti6Al4V increases dramatically, while the addition of Y_2_O_3_ suppressed the oxidation process.
(3) The XRD results indicated that the intensity of the diffraction peaks on the surface phase of TiO_2_ and Al_2_O_3_ after oxidation at different temperatures. The oxidation kinetics of the Ti6Al4V and Ti6Al4V-1.0 wt.% Y_2_O_3_ samples obeyed parabolic law between 600 °C and 800 °C and at 1000 °C followed a linear law.
(4) The microhardness values of Ti6Al4V increase continuously at 600 °C, 800 °C, and 1000 °C. However, the surface microhardness value of the Ti6Al4V-1.0 wt.% Y_2_O_3_ alloy increases until 800 °C and then decreases at 1000 °C. The change in microhardness was mainly in the oxide surface layer hardening.

## Figures and Tables

**Table 1:** The cross-sectional thickness of Ti6Al4V and Ti6Al4V-1.0 wt.% Y_2_O_3_ samples oxidized at different temperatures.

Sample	600 °C	800 °C	1000 °C
Ti6Al4V	59.55 μm	69.84 μm	139.15 μm
Ti6Al4V-1.0 wt.% Y_2_O_3_	35.73 μm	63.93 μm	80.34 μm

## Data Availability

Data will be made available on request.
